# The Harris hip score: Do ceiling effects limit its usefulness in orthopedics? A systematic review

**DOI:** 10.3109/17453674.2010.537808

**Published:** 2010-11-26

**Authors:** Kim E Wamper, Inger N Sierevelt, Rudolf W Poolman, Mohit Bhandari, Daniël Haverkamp

**Affiliations:** ^1^Department of Orthopaedic Surgery, Academic Medical Centre, Amsterdam; ^2^Department of Orthopaedic Surgery, OLVG, Amsterdam, the Netherlands; ^3^Division of Orthopaedic Surgery, Departments of Surgery and of Clinical Epidemiology and Biostatistics, McMaster University Medical Centre, Hamilton, ON, Canada

## Abstract

**Background and purpose:**

The Harris hip score (HHS), a disease-specific health status scale that is frequently used to measure the outcome of total hip arthroplasty, has never been validated properly. A questionnaire is suitable only when all 5 psychometric properties are of sufficient quality. We questioned the usefulness of the HHS by investigating its content validity.

**Methods:**

We performed a systematic review based on a literature search in PubMed, Embase, and the Cochrane Library for descriptive studies published in 2007. 54 studies (59 patient groups) met our criteria and were included in the data analysis. To determine the content validity, we calculated the ceiling effect (percentage) for each separate study and we pooled data to measure the weighted mean. A subanalysis of indications for THA was performed to differentiate the populations for which the HHS would be suitable and for which it would not. A ceiling effect of 15% or less was considered to be acceptable.

**Results:**

Over half the studies (31/59) revealed unacceptable ceiling effects. Pooled data across the studies included (n = 6,667 patients) suggested ceiling effects of 20% (95%CI: 18–22). Ceiling effects were greater (32%, 95%CI:12–52) in those patients undergoing hip resurfacing arthroplasty.

**Interpretation:**

Although the Harris hip score is widely used in arthroplasty research on outcomes, ceiling effects are common and these severely limit its validity in this field of research.

In evidence-based medicine, the use of clinically important patient outcomes in clinical research is paramount (Wright and Young 1997, Shi et al. 2009). Quality of life and function are usually measures that are important for patients and healthcare providers. The Harris hip score (HHS) is one such measure that has frequently been used to measure outcome after total hip arthroplasty (Haverkamp et al. 2008).

Harris (1969) developed this score with a rating scale of 100 points and with domains of pain, function, activity, deformity, and motion. It was designed for use in young men with often long-standing severe secondary osteoarthritis after a fracture of the acetabulum that was operated on with a Smith-Petersen vitallium mold arthroplasty. Although not originally designed for hip arthroplasty (THA) patients, it is widely used for this population. Since its introduction, several authors have reported the score to be a valid outcome measure for THA based on good construct validity alone (Harris 1969, Soderman and Malchau 2001, Shi et al. 2009). While construct validity is important, it is not the sole factor in evaluating the overall validity of an outcome questionnaire. Reliability, internal consistency, content validity, and responsiveness are also important. A questionnaire is suitable only when all 5 psychometric properties are of sufficient quality (Terwee et al. 2007).

Content validity assesses the extent to which a metric measures all aspects of a certain phenomenon. The amount of ceiling and floor effects present determine the quality of the content validity. A floor effect occurs when several of the patients score the lowest possible score, whereas a ceiling effect occurs when several of the patients score the highest possible score.

Given the persistent use of the HHS in clinical research, we systemically reviewed clinical trials of primary hip arthroplasty using the HHS as an outcome measure. We hypothesized that ceiling effects are common, thereby limiting the validity of the HHS in arthroplasty outcome research.

## Methods

Our systematic review conformed to the PRISMA guidelines for reporting of meta-analyses and systematic reviews (Moher et al. 2009).

### Information sources and search

We performed a systematic review of the literature by performing a computerized literature search in PubMed, Embase, and the Cochrane Library for studies published in 2007, searching for “hip” and “arthroplasty” both as free text and as MESH terms.

### Eligibility criteria

We included all descriptive trials, both prospective and retrospective, reporting on the outcome of primary total hip arthroplasty. Our inclusion criteria for further analysis were: articles published in English, patients undergoing primary total hip arthroplasty, and a range (standard deviation) of the HHS score reported in the article.

### Study selection

2 of the authors (DH and KW) independently selected titles and abstracts for possible inclusion. Full-text manuscripts were retrieved for any abstracts that appeared potentially eligible. The final decision to include a paper was based on a consensus between the 2 reviewers.

### The data collection process

Data extraction was performed independently by the same 2 authors (KW and DH), after which the data were compared and a consensus obtained.

### Calculation of ceiling effects

Harris hip scores were reported as averages with SD or range. If no SD was given in the study, it was calculated from the range to estimate the percentage ceiling effect. A ceiling effect means that several patients score the highest possible score, thus they “reach the ceiling”. A ceiling effect is caused when the test items are not challenging enough for a group of individuals because the test has a limited number of difficult items or even an inappropriate item selection (McHorney and Tarlov 1995). It will lead to a shortcoming in the discriminative ability of the test to detect clinically relevant changes; a person may continue to improve, but the test does not capture that improvement. A floor or ceiling effect of 15% is considered the maximum acceptable (Terwee et al. 2007).

### Analysis

As described by Walter and Yao (2007), it is possible to estimate the SD from a study when the mean and range and the size of the population is known. This method is widely used and accepted. We used it to estimate the SD for those studies where only the range and size (n) were given. In this data calculation, we assumed a normal distribution in the patient populations. This allowed us to estimate the percentage of ceiling effect present. This was calculated for each separate study, and data were also pooled to calculate a weighted overall percentage for all patients.

We performed a subgroup analysis based on the indication for THA to assess whether the HHS would be suitable for any subgroup. Also, a subanalysis for the influence of age and length of follow-up on the content validity was performed. Finally, we conducted a subgroup analysis evaluating ceiling effect in patients who underwent hip resurfacing arthroplasties.

We report a descriptive analysis of the ceiling effects, as percentage with 95% confidence interval (CI). For analysis, we used the HHS score from the latest follow-up reported. A linear logistic regression was performed to search for factors influencing the percentage ceiling effect (indication, retrospective or prospective trial, average age, length of follow-up period, or type of procedure (hip resurfacing, minimally invasive techniques, or normal THA). Statistical analyses were performed using SPSS software version 15.0.

## Results

### Literature search

Of 764 potential studies, 54 studies—of which 59 groups could be reviewed (5 trials were comparative, describing 2 populations; altogether, 6,667 patients)—were suitable for inclusion. Figure shows the reasons for exclusion of certain studies. 45 studies reported primary THA for several indications, 13 of which focused on specific patient populations (4 osteonecrosis, 9 dysplasia). Hip resurfacing was reported in 4 studies. Details of each study included are given in the Table.

**Figure:**
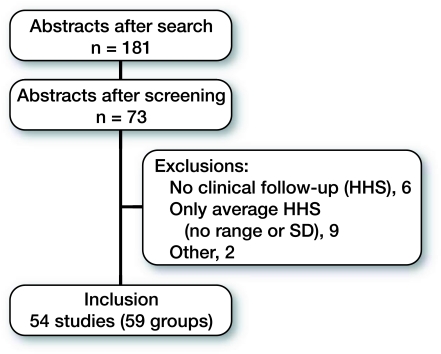
Flow diagram of data search

**Table:** Studies included

Authors	Year	Group	Follow–up in years	n	Average HHS	HHS range	SD	Ceiling effect (%)
Lim SJ et al.	2007	dysp	4.8	25	93.8	76–100	8.9	24
Kaneuji A et al.	2007	dysp	15.2	55	89	75–100	5.98	3
Incavo SJ et al.	2007	mix	6.7	143	91	63–100	10.64	20
Saito S et al.	2007	mix	6.4	76	95	91–100	1.89	1
Min BW et al.	2007	mix	7.7	98	92.9	83–99	^b^	0
Wangen H et al.	2007	dysp	13	49	88	62–100	11.44	15
León JL et al. #	2007	avn	2.9	24	**^a^**	**^a^**	**^a^**	0
Malizos KN et al.	2007	mix	5	245	94	69–97	**^b^**	0
Lin YC et al.	2007	mix	0.25	85	92	82–100	4.5 **^c^**	4
Zhang H et al.	2007	avn	1.9	72	92.4	78–100	6.05	10
Yates PJ et al.	2007	mix	11.1	122	86	47–100	15.6	18
Robinson RP	2007	mix	1	69	94	**^c^**	6 **^c^**	16
Robinson RP	2007	mix	1	92	92	**^c^**	11 **^c^**	23
Bragdon CR et al.	2007	mix	6.9	244	91.1	37–100	11.9 **^c^**	23
Flecher X et al.	2007	dysp	10.3	97	93	40–100	21.2	37
Le Duff MJ et al.	2007	mix	6.2	144	90.6	41–100	18.85	31
Le Duff MJ et al.	2007	mix	6.2	626	93.8	38–100	17.86	36
Lusty PJ et al.	2007	mix	6.5	259	95	61–100	11.56	33
Braun A et al.	2007	mix	6.5	37	91	33–100	27.3	37
Ochs U et al.	2007	mix	8.1	66	90.1	58.7–99.9	**^b^**	0
Kohler S et al.	2007	dysp	12.2	98	93	60–100	13.2	30
Leali A et al.	2007	mix	4.3	62	97	87–100	4.4	25
Hing CB et al.	2007	mix	3	227	95.2	47–100	16.39	38
Karatosun V et al.	2007	mix	3	71	93	64–100	12.18	28
Ender SA et al.	2007	mix	5	97	92	63–100	11.6	25
Vassan UT et al.	2007	mix	7	112	89	62–100	10.8	15
Kim YL et al.	2007	other	11	12	82.3	69–92	^b^	0
Lian YY et al.	2007	other	7.8	52	91.6	69–100	9.94	11
Parsch D et al.	2007	other	16	43	80	38–100	19.32	15
Guyen O et al.	2007	dysp	3.3	167	83.4	25–100	22.19	22
Akhavan S et al.	2007	dysp	6.2	99	98	86–100	4.8	34
Boyd HS et al.	2007	other	4.3	19	84	53–98	**^b^**	0
Fink B et al.	2007	mix	5.3	214	91.2	**^c^**	13.1 **^c^**	25
Isaac DL et al.	2007	mix	7.6	45	89	41–100	14.9 **^c^**	23
Lusty PJ et al.	2007	other	6.7	33	90	78–100	5.76	4
Baumann B et al.	2007	mix	9.5	69	85	**^c^**	13 **^c^**	12
Baumann B et al.	2007	mix	9.5	37	86	**^c^**	14**^c^**	18
Foucher KC et al.	2007	mix	1	28	95	61–100	17	38
Zhang XL et al.	2007	mix	1.5	27	94.5	92–96	^b^	0
Lachiewicz PF et al.	2007	mix	10.5	70	88	44–100	18.48	26
Mazoochian F et al.	2007	mix	7	10	94.4	85–100	4.1 **^c^**	9
Ito H et al.	2007	other	12	43	80.3	25–100	25.44	22
Nakamura Y et al.	2007	other	6.8	23	93.4	**^a^**	^a^	9
Yoon KS et al.	2007	mix	10.7	37	90	72–100	9.12	16
Yoon KS et al.	2007	mix	10.7	38	91	74–100	7.68	10
Cieliński Ł et al.	2007	mix	1	13	87.7	**^c^**	12 **^c^**	14
Kim YH et al.	2007	avn	11.2	194	91	59–100	11.52	22
Vidyadhara S et al.	2007	avn	4.1	45	96	**^c^**	3 **^c^**	9
Berend KR et al.	2007	mix	5	1080	88.3	**^c^**	8.3 **^c^**	8
Grübl A et al.	2007	mix	10	105	92	44–100	19.2	34
Kim KI et al.	2007	other	4.8	58	90	42–100	21.12	32
Harada Y et al.	2007	dysp	8.3	81	87.5	**^c^**	8.6 **^c^**	7
Jacob HA et al.	2007	mix	12	102	97	92–100	2.0	7
Ha YC et al.	2007	mix	5.5	74	94	82–100	5.04	12
Poggie RA et al.	2007	mix	1	157	93	51–100	15.96	33
Poggie RA et al.	2007	mix	1	315	92	36–100	19.04	34
Amstutz HC et al.	2007	dysp	6	59	92.5	41–100	22.66	37
Angin S et al.	2007	mix	3.8	8	95	82–100	9.5 **^c^**	30
Habermann B et al.	2006	other	11	15	89	76–100	7.49	7

**^a^** Described per patient.

**^b^** SD not calculated since range shows a ceiling effect of 0%. For instance, a range of 60–98 shows that the maximum score of 100 is not reached in this study; hence, the ceiling effect is 0%.

**^c^** SD given. dysp: hip dysplasia; mix: mixed group; avn: avascular necrosis of femoral head, fracture, osteotomy. etc.

### Ceiling effects

31/59 patient groups showed a ceiling effect greater than 15%. Pooling across the 59 patient groups showed a ceiling effect of 20% (CI: 18–22).

When studies needing a calculation of SD were excluded and only the 14 studies in which an SD was given were included, we found an average ceiling effect of 15.8% (4–30); 7 of the 14 studies had a ceiling effect of more than 15%.

In the studies evaluating total hip arthroplasty in patients with avascular necrosis of the femoral head, the ceiling effect averaged 16% (CI: 8–24). Similarly, in patients with total hip arthroplasty for osteoarthritis secondary to dysplasia, the ceiling effect averaged 24% (CI: 18–31). In patients treated with a hip resurfacing, the mean ceiling effect was 32% (CI: 12–52).

Indication, study design, patient age, length of follow-up, and type of procedure had no statistically significant influence on the magnitude of the ceiling effect (p-values all > 0.05).

## Discussion

### Key findings

Our review shows that the Harris hip score has frequent ceiling effects in trials evaluating outcomes of primary hip arthroplasty, which indicates that it has limited application in exploration of treatment differences using newer techniques.

### Strengths and limitations

Our meta-analysis has several strengths. We used a comprehensive systematic approach to identify relevant papers, we assessed the reliability of our assessments, and included a sufficient number of trials to be able to reach a conclusion. Finally, our systematic review followed the international PRISMA guidelines for reporting.

Our review does have some limitations. We calculated ceiling effect based on the assumption that scores in every population had a normal distribution. It is possible that normality was not met due to insufficient sample size, or merely because of the existence of the ceiling effect we were trying to investigate. However, in half of studies in which the SD was given rather than calculated by us, a ceiling effect was found. We determined only the ceiling effects and not floor effects because we believed that ceiling effects are the main limitations of the Harris hip score (Soderman and Malchau 2001, Kirmit et al. 2005). None of the studies that investigated the reliability included the floor score in the range of distribution, while most included the ceiling value of 100.

### Previous literature

In 1969, when the HHS was developed, it probably had excellent content validity due to the nature of the patient population and type of implant at that time (Harris 1969). However, indications for joint replacement have expanded over time and improvements in implant designs and techniques have led to improved outcomes. The ability of a functional outcome measure to distinguish clinically relevant improvements in outcomes with changes in prosthetic design is important. Ceiling effects in an instrument can hide these differences when patients already score the maximum possible score and cannot improve on that score. For example, a 75-year-old patient just able to walk 2 hours at a normal pace would have the same score as a 45-year-old patient who has returned to running marathons.

### Implications for future research

There are plenty of alternative scoring systems, including the WOMAC score, the Oxford 12-item questionnaire, and the HOOS (Roorda et al. 2004, Gosens et al. 2005, de Groot et al. 2009). Ostendorf et al. (2004) evaluated the WOMAC score and the Oxford 12-item questionnaire for validity. The Oxford hip score did well in their study on all validity items. De Groot et al. (2009) evaluated the content validity of the Dutch version of the hip disability and osteoarthritis outcome score (HOOS) and reported no ceiling effect and good validity.

Besides having a problematic ceiling effect, HHS includes a physician's physical examination component. Previous studies have shown that physical examination has a high intraobserver variablility (Poolman et al. 2009). Consequently, investigators have commonly used a modified Harris hip score without the physical examination part. Thus, the modified HHS suffers the drawbacks of ceiling effects as well as the problems of a non-validated modification of the original HHS score (Ragab 2003).

### Conclusion

Based on our systematic review and meta-analysis, we conclude that the Harris hip score commonly shows ceiling effects, which limit its usefulness in trials evaluating the efficacy of primary total hip arthroplasty.
